# Vulnerable neoatherosclerosis in coronary artery with whole circumference showing intravascular echo attenuation

**DOI:** 10.1002/ccr3.2152

**Published:** 2019-04-23

**Authors:** Yukiho Hirota, Tetsuya Nomura, Kenshi Ono, Yu Sakaue, Daisuke Ueno, Yusuke Hori, Kenichi Yoshioka, Masakazu Kikai, Natsuya Keira, Tetsuya Tatsumi

**Affiliations:** ^1^ Department of Cardiovascular Medicine Kyoto Chubu Medical Center Nantan City Kyoto Japan

**Keywords:** echo attenuation, neoatherosclerosis, percutaneous coronary intervention

## Abstract

Neoatherosclerosis is emerging as a stent‐associated problem that has not yet been fully resolved. Because in‐stent restenosis with a neoatherosclerotic etiology is associated with a high risk of acute coronary syndrome and a poor survival prognosis, it is essential to precisely identify patients at risk using advanced imaging modalities.

## 1 INTRODUCTION

Percutaneous coronary intervention with stent deployment is widely performed throughout the world for the treatment of ischemic heart disease. Drug‐eluting stents (DES) are predominantly used due to their features that minimize the limitations of bare metal stents (BMS). However, critical issues remain regarding late complications such as in‐stent restenosis (ISR) and late‐stent thrombosis. One of their causes is neoatherosclerosis, which is a form of accelerated atherosclerosis that occurs within stented segments.

## 2 CASE REPORT

A 64‐year‐old man was admitted to our hospital for further examination regarding ischemic findings on myocardial perfusion scintigraphy. His cardiovascular risk factors were type 2 diabetes mellitus and dyslipidemia. His blood pressure was 127/71 mm Hg, and pulse was 89/min and regular. His body mass index was 18.1. No particular finding was noted on physical examination, and laboratory data showed no abnormality without hyperglycemia (blood sugar: 251 mg/dL). No significant sign of cardiac ischemia was noted on twelve‐lead electrocardiography. Chest X‐ray showed a normal cardiothoracic rate (42.8%) and no abnormal pulmonary lesion. The left ventricular wall motion was within normal limits and no apparent valvular disorder was seen on ultrasound echocardiography.

**Figure 1:**
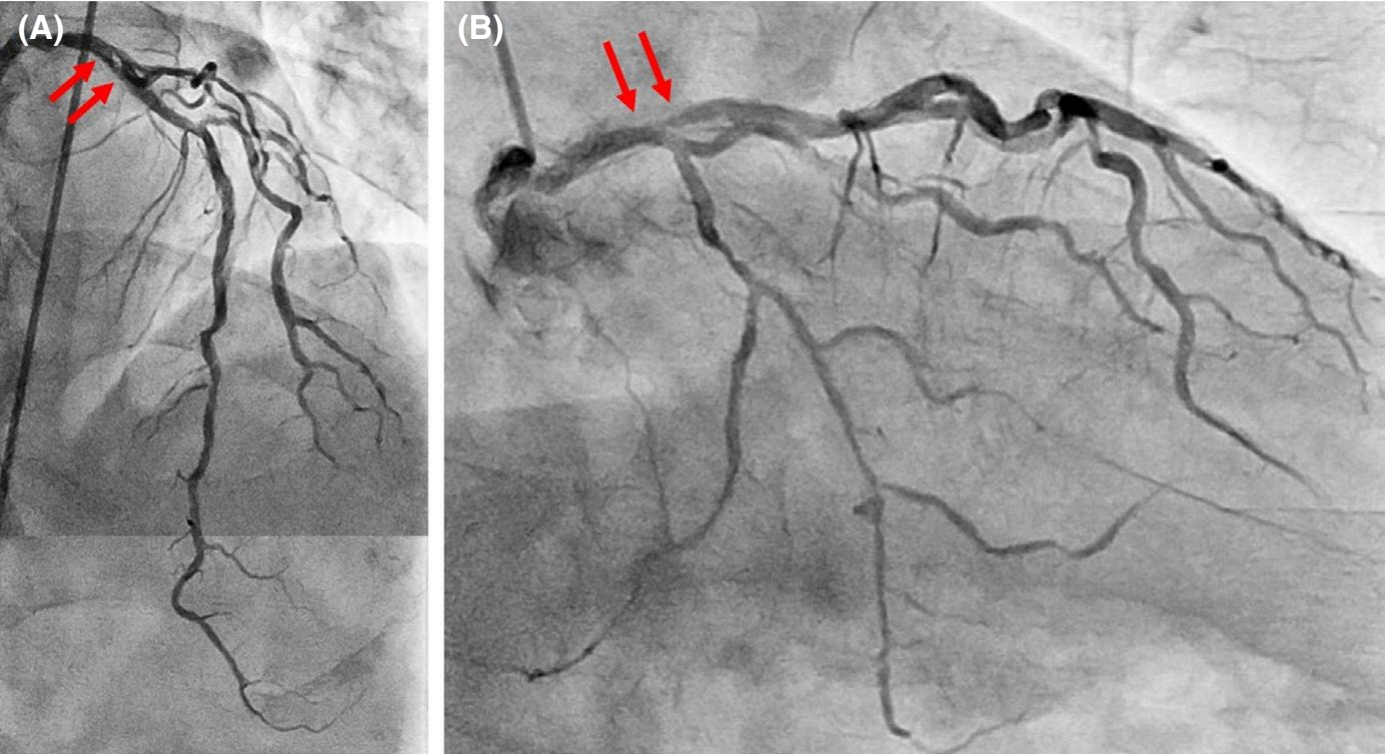
A control image of the left coronary artery. Arrows indicate a moderate ISR at the ostium of the LAD artery. A, Left anterior oblique and cranial view. B, Right anterior oblique and caudal view

**Figure 2:**
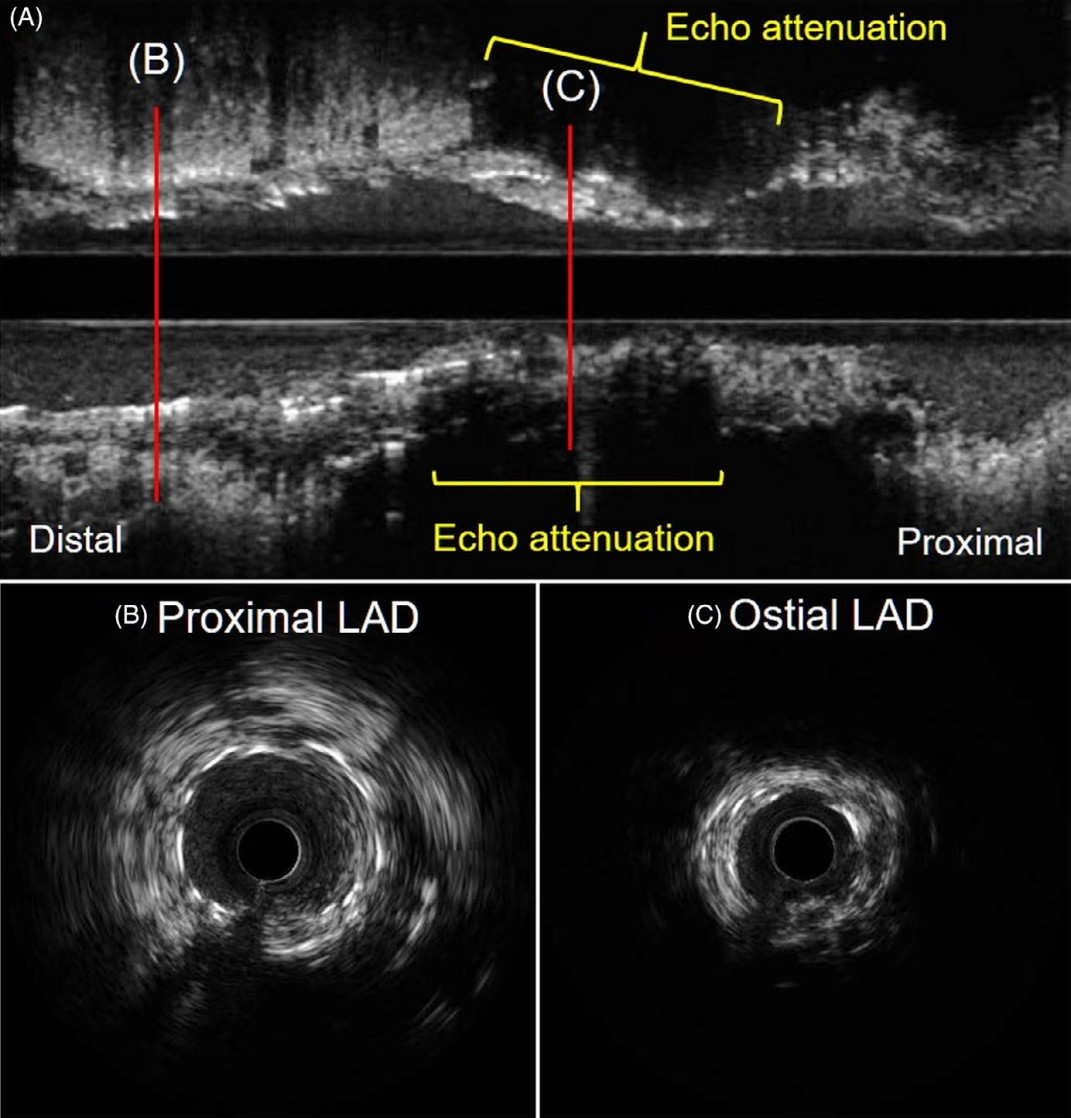
IVUS findings from the proximal LAD artery to the left main coronary artery. A, Longitudinal image. B, Cross‐sectional image at proximal LAD artery. C, Cross‐sectional image at ostial LAD artery. Typical echo attenuation of whole circumference was observed at the stenosed segments

**Figure 3:**
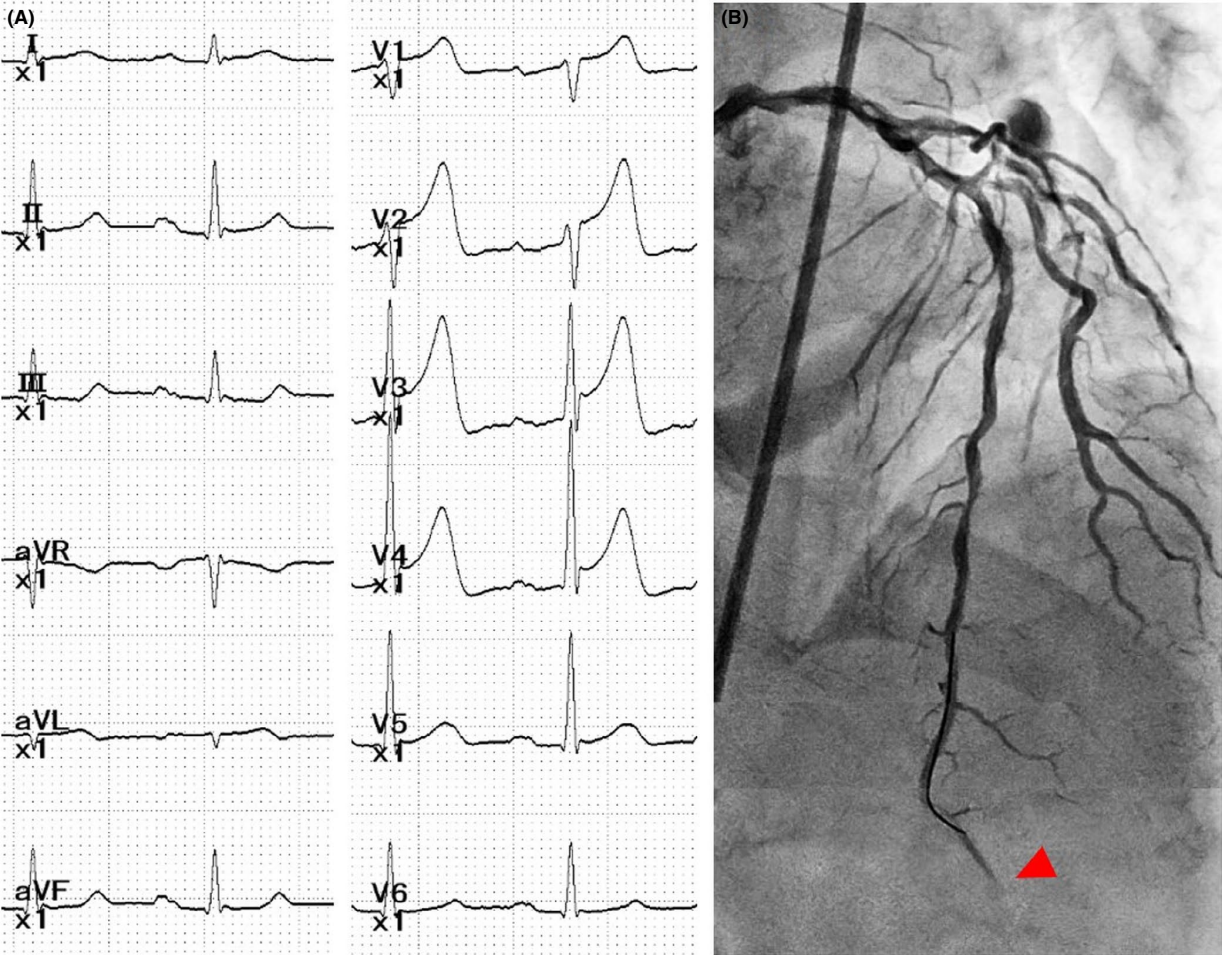
A, Electrocardiography showing ST‐segment elevation with increased voltage of T waves in V2‐4 leads during chest symptoms. B, Flow limitation at the distal end of the LAD artery (Arrowhead)

**Figure 4:**
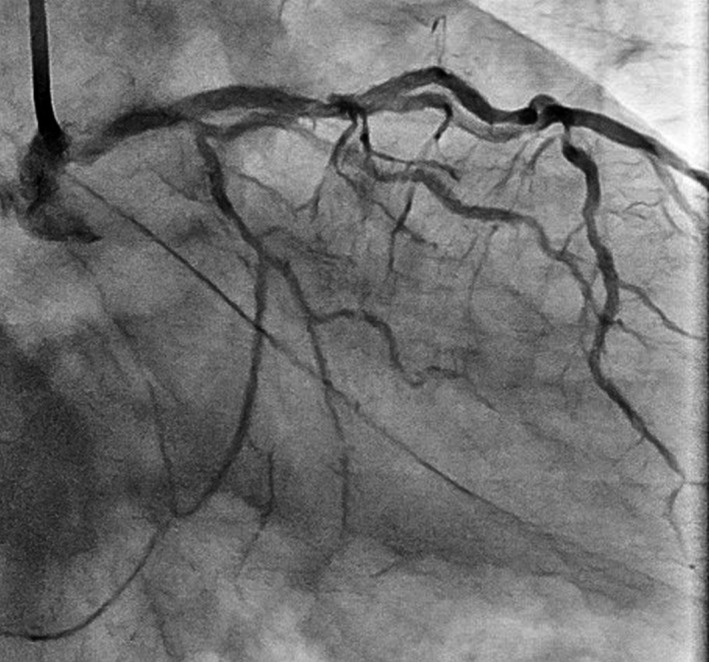
Final angiography showing favorable vascular expansion with no flow disturbance in the LAD artery

Coronary angiography showed moderate in‐stent restenosis (ISR) at the ostium of the left anterior descending (LAD) artery (Figure 1A,B Arrows). A Cypher stent (Cordis, CA, USA) had been implanted there 12 years previously. We first checked the lesion with OptiCross (Boston Scientific) intravascular ultrasound (IVUS). The implanted Cypher stent segmentally exhibited less expansion than expected (Figure 2C). A diffuse longitudinal distribution of high‐echoic plaque partially with ambiguous stent struts and typical echo attenuation of the whole circumference were observed in the stenosed segments (Figure 2A,C). Just after the IVUS scan, the patient suddenly complained of severe chest pain, and ST‐segment elevation with an increased voltage of T waves in V2‐4 leads appeared on electrocardiography (Figure 3A). Coronary angiography showed stasis of blood flow at the distal end of the LAD artery (Figure 3B, Arrowhead). We suspected distal embolism, and the injection of a vascular relaxant agent after aspirating the blood in the LAD artery favorably recovered blood flow. Then, using a Filtrap catheter (NIPRO), a filter device to prevent distal embolisms, we performed balloon angioplasty with a scoring balloon catheter several times and finally inflated a drug‐coated Sequent Please balloon catheter (NIPRO) for one minute. After successful retrieval of the Filtrap catheter, we noted favorable vascular expansion with no flow disturbance in the LAD artery (Figure 4). Postoperatively, no significant myocardial damage, which would be indicated by an increased level of creatinine phosphokinase (CPK), was observed.

## 3 DISCUSSION

Neoathrosclerosis is a stent‐associated problem that has not yet been fully resolved.^1^, ^2^ Chronic inflammation and insufficient functional endothelialization induce neoatherosclerosis inside both bare metal stents (BMS) and drug‐eluting stents (DES), causing late stent failure such as ISR or thrombosis following plaque rupture. Therefore, it is essential to precisely identify those patients at risk while conducting PCI.^3^

Neoatherosclerosis is usually confirmed by advanced imaging modalities including optical coherence tomography (OCT) and IVUS. OCT analysis in patients with DES‐ISR demonstrated a wide variety of abnormal findings, such as thin cap fibroatheroma‐containing neointima, in‐stent neointimal rupture, and intraluminal thrombi.^4^ Neoatherosclerosis has been reported to be more frequently observed and exhibit more longitudinal extension in BMS compared with DES.^5^ On the other hand, lipid‐rich neoatherosclerosis is considered to develop inside stents earlier in DES than BMS.^6^

Intravascular ultrasound is also one of the useful imaging modalities to evaluate neoatherosclerosis. IVUS findings such as echo attenuation, the intraplaque echolucent zone, and spotty calcification have been reported to be associated with instability of coronary artery disease. Moreover, echo‐attenuated plaque, especially superficial echo attenuation, was the most reliable IVUS sign for identifying high‐risk plaque.^7^ On pathological analysis, necrotic cores or lipid pools are usually observed at the site of echo attenuation. Therefore, the arc of echo attenuation on IVUS is significantly correlated with the arc of the histopathologic lipid/necrotic core burden. That is, the extent of the arc of echo attenuation on IVUS may be proportional to the instability of the plaque.

Although there was less severe stenosis at the ostium of the LAD artery in our case, mere IVUS passage through the lesion caused embolization in the distal segment of the LAD artery, which is highly suggestive of vulnerability of the plaque at the target lesion. IVUS assessment at that segment showed superficial echo attenuation in part and echo‐attenuated plaque on the whole circumference. These IVUS findings and the episode of initial embolization encouraged us to use a device to prevent the further occurrence of distal embolization following the procedure.

We completed our procedure by inflating a drug‐coated balloon (DCB) catheter without additional DES deployment. DCB is highly efficient for patients presenting with ISR. DCB use markedly inhibits late neointimal formation. A previous study demonstrated how DCB potentially provides an attractive therapeutic strategy for neoatherosclerosis.^8^ However, another study reported that neoatherosclerosis can develop after DCB treatment for ISR.^9^

## 4 CONCLUSION

Here, we described a patient with vulnerable neoatherosclerosis in the coronary artery showing intravascular echo attenuation of the whole circumference. Although the phenomenon of neoatherosclerosis has not yet been fully elucidated, we propose that a more extensive distribution of echo‐attenuated plaque longitudinally and circumferentially may be a critical sign of vulnerability to neoatherosclerosis.

## CONFLICTS OF INTEREST

No author has a conflict of interest.

## AUTHOR CONTRIBUTION

HY: involved in study conception, design, drafting of manuscript, and the primary doctor for the patient; TN: involved in drafting manuscript and the corresponding author. KO, YS, DU, and YH: performed data acquisition. KY: was one of the main doctors. MK: involved in study conception and design, and performed critical revision of this report. NK: was a supervisor. TT: performed critical revision of this report.
